# Unique degeneration signatures in the cerebellar cortex for spinocerebellar ataxias 2, 3, and 7

**DOI:** 10.1016/j.nicl.2018.09.026

**Published:** 2018-09-27

**Authors:** Carlos R. Hernandez-Castillo, Maedbh King, Jörn Diedrichsen, Juan Fernandez-Ruiz

**Affiliations:** a CONACYT - Instituto de Neuroetologia, Universidad Veracruzana, Xalapa, Mexico; b Brain and Mind Institute, Western University, London, ON, Canada; c Department of Psychology, University of California, Berkeley, CA, USA; d Department of Computer Science and Department of Statistical and Actuarial Sciences, Western University, London, ON, Canada; e Departamento de Fisiologia, Facultad de Medicina, Universidad Nacional Autonoma de Mexico. Mexico

**Keywords:** Spinocerebellar ataxia, Degeneration, VBM, Atrophy, Classification

## Abstract

Spinocerebellar ataxias (SCAs) are a heterogeneous group of neurodegenerative diseases that selectively affect vulnerable neuronal populations in the cerebellum and other subcortical regions. While previous studies have reported subtype differences in the absolute amount of degeneration in specific regions of interest, they failed to account for two important factors. First, they did not control for overall differences in the severity of the degeneration pattern, and second, they did not fully characterize the spatial pattern of degeneration for each SCA subtype. Here, we provide a systematic characterization of the spatial degeneration patterns for three polyQ SCAs (55 patients, either SCA2, SCA3, or SCA7) while controlling for the severity of the degeneration pattern. After this correction, the cerebellar degeneration pattern can successfully classify between the three different SCA subtypes with high cross-validated accuracy. Specifically, degeneration in SCA3 disproportionally affects motor regions of the cerebellar cortex, which explains the relatively severe motor symptoms observed in this subtype. Our results demonstrate that each of the three studied SCA subtypes has a unique cerebellar degeneration signature, hinting at differences in the disease process. Clinically, these differentiable patterns of cerebellar degeneration can be used to reliably discern subtypes, even at relatively early stages of the disease.

## Highlights

• Anatomical degeneration in different SCA subtypes show considerable overlap.
• We analyzed degeneration pattern independently of degeneration severity.
• The pattern allowed subtype classification with on average 92% accuracy.
• Differences in degeneration patterns related to differences in behavioral symptoms.

## 1 Introduction

Neurodegenerative diseases are debilitating conditions that result in the progressive death of nerve cells that can affect motor, sensory, and cognitive systems. Spinocerebellar ataxias (SCAs) are a heterogeneous group of neurodegenerative diseases, primarily characterized by clinical manifestations of ataxic gait, dysarthria, dysmetria, and dysdiadochokinesia (Manto, 2005; Sun et al., 2016). To date, 40 SCAs have been characterized (SCA1 – SCA40) and amongst these, twenty-eight have gene descriptions that denote their genetic abnormalities, while the other twelve are diagnosed by their specific symptomatology alone (Manto, 2005; Sun et al., 2016). The most common subtypes, also known as polyQ SCAs (SCA1, SCA2, SCA3, SCA6, SCA7, SCA17 and DRPLA), are caused by the expansion of a CAG repeat sequence located within the coding region of specific genes. This expansion leads to an abnormally long polyglutamine tract in the encoded proteins, which progressively affects the cerebellar cortex and the deep cerebellar nuclei (Manto, 2005; Dueñas et al., 2006; Sun et al., 2016).

Early neuropathological studies on SCA identified severe neuronal loss in the cerebellar cortex and in brainstem structures (Orr et al., 1993; Soong and Paulson, 2007; Yamada, 2013). Recent improvements in neuroimaging techniques have made it possible to map the degenerative processes in vivo. Different methods, including volumetry and voxel-based morphometry (VBM), have been used to study the patterns of brain tissue atrophy in several SCA subtypes (Pulst et al., 1996; Schulz et al., 2010; Goel et al., 2011; D'Abreu et al., 2012; Hernandez-Castillo et al., 2013, Hernandez-Castillo et al., 2015, Hernandez-Castillo et al., 2017; Jacobi et al., 2013; Reetz et al., 2013; Fahl et al., 2015; de Rezende et al., 2015; for a recent review see Klaes et al., 2016).

Although these studies have suggested differentiable patterns of degeneration, a careful quantitative comparison of degeneration patterns for different subtypes is still missing. Previous studies have been limited by two factors. First, most of these studies have used manual segmentation to calculate the total volume of pre-defined regions-of-interest (ROIs) (Klaes et al., 2016). The specific ROI's (cerebellar lobules, hemisphere, vermis, etc) differ between those studies, making it difficult to compare and integrate the results across them. Furthermore, the use of ROIs obscures any variation of degeneration within the chosen regions. The cerebellum is typically parcellated into cerebellar lobules (i.e, Kansal et al., 2016), and this lobular nomenclature is mostly sensitive to anterior-to-posterior differences, overlooking any potential medial-to-lateral variation within each lobule (King et al., 2017).

Second, previous studies have not systematically disentangled the *severity* of degeneration from the *pattern* of degeneration. *Severity* can be defined as the average amount of degeneration within a particular structure while the *pattern* represents the relative distribution of degeneration within that structure. The rate of disease progression may differ substantially between subtypes. In addition, the first behavioral symptoms may occur at different points over the course of the disease. Therefore, patients with particular subtypes may seek medical attention at earlier stages of the degeneration process. Both of these factors could yield differences in the overall amount of degeneration reported across subtypes. Differences in the absolute amount of degeneration in specific ROI's, as reported by previous studies, could result in differences in the overall amount of degeneration, or they could be indicating differential patterns of degeneration. To overcome this problem, it is necessary to determine whether the spatial distribution of the degeneration (for a comparable stage in the disease) differs across subtypes.

Therefore, we aimed to systematically characterize patterns of degeneration for three polyQ SCAs. Our approach diverges from previous studies in two major ways. First, we measured the pattern continuously across the surface of the cerebellum, rather than segmenting the cerebellum into pre-defined ROIs (e.g., Kansal et al., 2016). Second, for each patient we accounted for the overall severity of degeneration to emphasize the spatial pattern of degeneration in the cerebellar cortex. Each SCA subtype is the result of one specific gene mutation (Everett and Wood, 2004; Orr and Zoghbi, 2007) and if these genetic differences result in focally selective neurodegeneration, then the pattern of gray-matter degeneration in each SCA subtype should be differentiable across subtypes, even at early stages of the disease. If successful, this approach would provide a novel way of classifying different SCA subtypes based solely on patterns of cerebellar degeneration. Furthermore, by identifying the areas specifically impacted by each SCA subtype, we glean an insight into the neuronal populations that are most susceptible to subtype-specific mutations. Finally, the measures provide a possible biomarker for the progression of each disease and can be used to sensitively evaluate the effectiveness of future treatments.

## 2 Materials and methods

### 2.1 Subjects

The dataset used in this study was previously reported in separate studies of SCA2 (Hernandez-Castillo et al., 2015), SCA3 (Hernandez-Castillo et al., 2017), and SCA7 (Hernandez-Castillo et al., 2013). The final sample (55 patients) included 14 SCA2 (9 female), 17 SCA3 (10 female), and 23 SCA7 (11) patients. Fifteen age-matched volunteers (7 female) comprised the control group. General characteristics of the participants are presented in Supplementary Table 1. Neither patients nor controls had a history of any neurological disease, other than the SCA in the patient group, or any neuropharmacological treatment, and there was no significant difference in age between the patient and control groups (two-group *t*-test, *p* = .82) or between any patient group (ANOVA, F_(2,52)_ = 0.22, *p* = .88). All participants gave their informed consent before entering the study and all procedures in this study were conducted in accordance with the international standards dictated by the Helsinki Declaration of 1964. The procedures were carried out in accordance with the ethical standards of the committees on human experimentation of the Universidad Nacional Autonoma de Mexico.

### 2.2 Clinical assessment

Ataxia severity was measured using the Scale for the Assessment and Rating of Ataxia (SARA). The SARA score is comprised of eight items, including tests of gait, stance, sitting, and speech, as well as the finger-chase test, finger-nose test, fast alternating movements, and heel-shin test (Schmitz-Hübsch et al., 2006). The score ranges from 0 to 40, where a higher score indicates a greater impairment.

### 2.3 Image acquisition

All anatomical MRI images were acquired using a 3.0-T Achieva MRI scanner (Phillips Medical Systems, Eindhoven, Holland). The anatomical acquisition consisted of a 3-D T1 Fast Field-Echo sequence, with TR/TE of 8/3.7 ms, FOV of 256 × 256 mm; and an acquisition and reconstruction matrix of 256 × 256, resulting in an isometric resolution of 1 × 1 × 1 mm. To reduce patients' movements and prevent motion artifacts, participants were fitted with head pads and fastened with a body sheet to ensure optimal comfort. Brain images for each participant were checked by an expert radiologist to verify that artifacts, which are classically observed in excessive motion cases, were absent.

### 2.4 Cerebellar normalization and voxel-based morphometry

For each anatomical image, the cerebellum was isolated from the rest of the brain and normalized to the spatially unbiased infratentorial template of the cerebellum (Diedrichsen, 2006) using the SUIT toolbox in SPM12 (http://www.diedrichsenlab.org/imaging/suit.htm). The SUIT toolbox (v3.2) uses the segmentation algorithm implemented in SPM12 to create tissue probability maps of cerebellar gray-matter, cerebellar white-matter, and their cortical counterparts. The sum of cerebellar gray and white matter maps served as the cerebellar isolation mask. These isolation masks were individually checked and manually corrected using MRIcron software (http://people.cas.sc.edu/rorden/mricron/index.html). Normalization was performed using the diffeomorphic anatomical registration (DARTEL) algorithm (Ashburner, 2007), which deforms the cerebellum to fit the probability maps of cerebellar gray and white matter to the SUIT atlas.

We then employed voxel-based morphometry (VBM, Ashburner and Friston, 2000) to measure the amount of degeneration across the cerebellar cortex. Because the normalization was performed on cerebellar structures only, any changes in cerebellar volume could manifest either in the linear and non-linear component of the deformation. We therefore applied both components to the gray-matter probability map from each individual participant. The gray-matter probability was modulated to correct for volume changes during the spatial normalization step (Good et al., 2001) – i.e. the image intensities were scaled by the amount of contraction or expansion that had occurred during spatial normalization. The resultant image therefore served as an estimate of the relative amount of cerebellar gray-matter found in the individual volume corresponding to a single voxel in the atlas template.

### 2.5 Adjustment for brain-size and degeneration severity

The cerebellar volume is dependent on the overall size of the brain. To correct for this factor, we regressed out the volume of the cerebrum (including gray-matter, white-matter and cerebrospinal-fluid compartments) from the cerebellar volume obtained from SUIT. The ratio of the predicted values from this regression and the group averaged cerebellar volume was taken as a correction factor. We multiplied the individual's gray-matter map with this factor to obtain the scaled gray-matter intensity maps for the cerebellar cortex.

As a next step, we created individual maps that estimated the amount of local degeneration. For this, we used the scaled maps to calculate a gray-matter intensity map for the control group. We then subtracted the individual gray-matter intensity map of each patient from the average gray-matter intensity map of the control group. Finally, we created a version of these degeneration maps that was corrected for overall degeneration severity. For each patient, the average degeneration intensity across all gray-matter voxels was calculated, resulting in a measure of degeneration severity. We then adjusted for the degeneration severity by dividing the degeneration maps by the corresponding measure of degeneration severity. By applying this correction, we obtained an adjusted map that captured which regions demonstrated more than the average (values >1) or less than the average degeneration (values<1). The mean degeneration severity was also used to evaluate group differences and the relationship to motor symptoms, as measure by the SARA score.

### 2.6 Dentate and pontine regional analysis

The cerebellar cortex is not the only brain region affected in many spinocerebellar ataxias. Several reports have shown degeneration in the deep cerebellar and brainstem nuclei in the three subtypes that we study here (Della et al., 2008; D'Abreu et al., 2012; Hernandez-Castillo et al., 2016). To assess the amount of degeneration in these key areas, we defined ROI's for the dentate, pontine, and inferior olive based on the SUIT atlas definitions. Following the same procedure applied to the cerebellar GM, we used VBM to estimate the overall tissue volume (combining gray and white matter) in these ROI's. We then corrected for head size and calculated a metric of degeneration for each individual patient and ROI.

### 2.7 Multi-dimensional scaling

We used a multi-dimensional scaling approach (MDS; Torgerson, 1952) to visualize the separability of the degeneration patterns in cerebellar cortex. The MDS algorithm aims to place the degeneration pattern of each subject in a low-dimensional space, such that the between-subject distances in the original space (number of dimensions = number of voxels) are preserved as much as possible. In our application, we chose a two-dimensional space that best represented the differences between the degeneration groups.

### 2.8 Pattern classification

To validate the separability of the degeneration patterns, we used linear discriminant analysis (LDA; Fisher, 1938). Using a subset of the data (training set), we trained classifiers to 1) differentiate each SCA subtype from the control group, and 2) to differentiate between each pair of SCA subtypes. The predictive power of the classifier was then measured on an independent part of the data (testing set). The scaled, but otherwise uncorrected individual degeneration maps, were submitted to the classifier to conduct a binary classification between the control group and each SCA group (HC vs SCA2, HC vs SCA3, HC vs SCA7). Then, the uncorrected and severity-corrected individual degeneration maps were submitted to conduct binary classification between all SCA subtypes (SCA2 vs SCA3, SCA2 vs SCA7 and SCA3 vs SCA7). We hypothesized that the degeneration maps corrected for overall severity would result in higher classification accuracies, as the main difference should be attributable to the pattern, rather than the severity of the degeneration. Classification accuracies were calculated using leave-one-out cross-validation, each time leaving out one individual from each group, testing each possible pair of subjects. The proportion of overall correct classifications, and for each individual patient were recorded.

### 2.9 Analysis of average degeneration pattern

The corrected images for each patient group were then averaged separately for the three subtypes. For visualization purposes, the resultant maps were then converted into a surface representation using the SUIT flatmap (Diedrichsen et al., 2015). The surface representation of the cerebellum is designed to display cerebellar data in an easy-to-interpret two-dimensional space. To identify the sub-regions related to the ataxia impairment, we calculated the Pearson's correlation between the SARA score and the scaled gray-matter intensity maps from all SCA patients. Finally, to compare if each subtype had more or less degeneration in the ataxia-related regions, we measured the degeneration intensity for each patient in the regions demonstrating higher correlations. Patients' gray-matter intensity values were divided into subtypes for statistical comparison.

## 3 Results

The primary aim of this paper was to provide a detailed and quantitative comparison of the cerebellar degeneration patterns across three SCA subtypes. The quantification of such differences can provide important insights into differences underlying the disease process. To test whether such differences are actually present, we determined whether it would possible to distinguish between different SCA subtypes based on the degeneration pattern alone. If successful, this method would also provide a clinical tool to distinguish among subtypes. This innovation would be especially important for subtypes that are not identifiable using genetic markers.

### 3.1 Overall degeneration severity

**Fig. 1:**
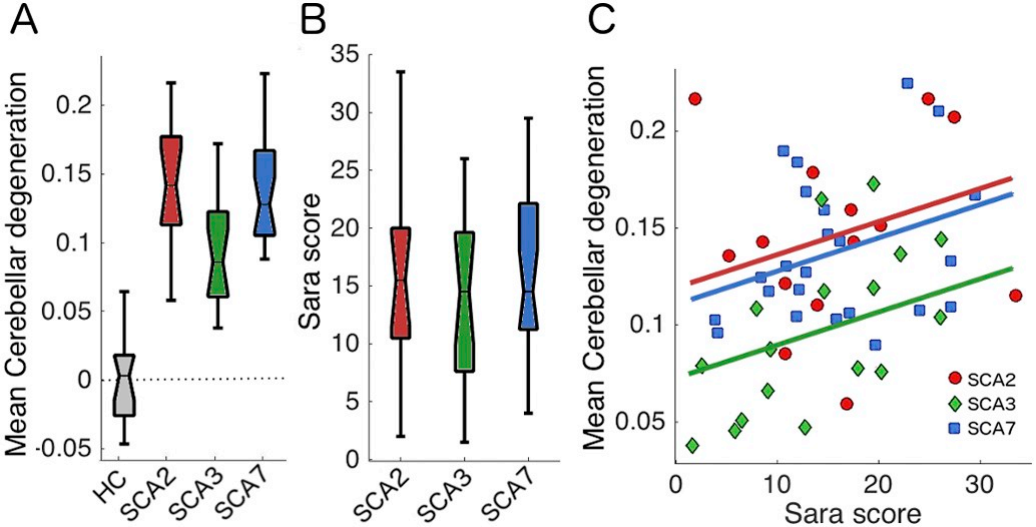
Cerebellar degeneration severity for healthy controls (HC) and each SCA group. A) Mean degeneration severity, the proportion of volume reduction relative to the mean of the controls, corrected for overall brain size. B) SARA score, a measure of the severity of ataxic symptoms, for each SCA group. A score of zero indicates no impairment. C) Relationship of mean degeneration of individual patients from different SCA subtypes as a function of the SARA score (see methods). The zero value in the y-axis indicates the average gray-matter probability (intensity) of the control group (no degeneration), 0.15 would indicate a 15% reduction in gray matter volume.

The overall severity of degeneration can be a confounding factor if volume reductions are directly compared in particular ROI's across SCA subtypes. Therefore, we first compared the overall amount of degeneration across the cerebellar cortex after correcting for overall brain size (see methods). As can be seen in Fig. 1a, degenerative processes in our sample were more pronounced in SCA2 and SCA7 than in SCA3. An ANOVA between the three patient groups yielded a significant omnibus test, (F_(2,52)_ = 7.145, *p* = .001). Therefore, the direct comparison of mean degeneration maps from different groups, without a correction for the degeneration severity, would heavily reflect these mean differences in disease severity instead of the differences in the pattern of degeneration.

### 3.2 Motor Impairment

The differences in degeneration severity would predict that SCA3 patients exhibit less severe behavioral symptoms as compared to the other two groups. The SARA scores, however, were similar across subtypes (Fig. 1b), as shown by the non-significant between-group ANOVA for the SARA score (F_(2,52)_ = 0.478, *p* = .622). There was a systematic relationship between the SARA score and the degeneration severity, such that patients with higher deficit scores showed more degeneration (*r* = 0.483, *p* = .001). Even after accounting for the SARA score, SCA3 patients still showed less degeneration than the other groups. To show this, we conducted an ANCOVA, using the SARA score as a covariate, which still revealed significant differences between SCA3 and the other two subtypes (F_(2,50)_ = 6.437, *p* = .003). This observation suggests that SCA3 patients exhibit heightened motor symptoms for an equivalent amount of cerebellar degeneration relative to SCA2 and SCA7 patients. This offers one possible explanation for the group differences in degeneration severity: the earlier onset of visible motor symptoms in the SCA3 group may have led them to seek medical attention earlier, and therefore made inclusion into our current sample more likely.

### 3.3 Degeneration in pontine, dentate and inferior olive

**Fig. 2:**
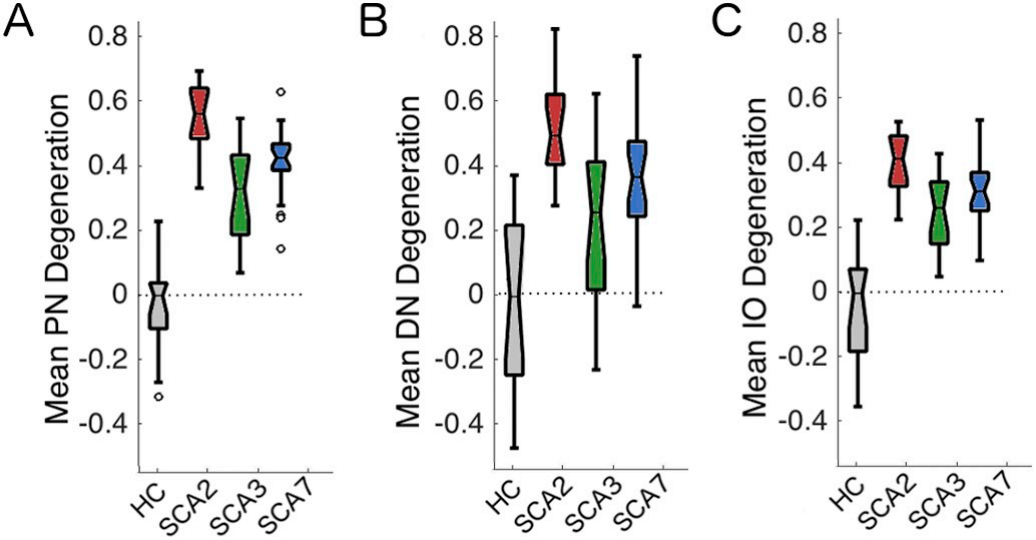
Brainstem degeneration severity for healthy controls (HC) and each SCA group. A) pontine (PN), B) dentate (DN) and C) Inferior Olive (IO).

One possible reason for the early symptom onset in the SCA3 subtype compared to the other two subtypes could be due to an earlier degeneration of brainstem and/or deep cerebellar region. Patients in the SCA3 group could have had more degeneration in the brainstem compared to patients with SCA2 and SCA7. This could possibly explain the early symptom onset even if the cerebellar cortex was less degenerated than in the other 2 subtypes. Therefore, we assessed the combined GM/WM volume of three input/output nuclei, which are normally affected in these three SCAs: the inferior olive, pontine, and dentate nuclei. In all three areas, however, SCA3 patients showed significantly less degeneration in comparison to SCA2 and SCA7 (Fig. 2). This observation suggests that the amount of degeneration in the brainstem and deep cerebellar ROIs was similar to the amount of degeneration in the cerebellar cortex. Hence, it cannot be the reason for the difference in symptom onset between these groups. Therefore, the most plausible explanation for the difference in ataxia impairment is that different areas of the cerebellar cortex are affected across subtypes.

### 3.4 Separability of degeneration patterns in the cerebellar cortex

**Fig. 3:**
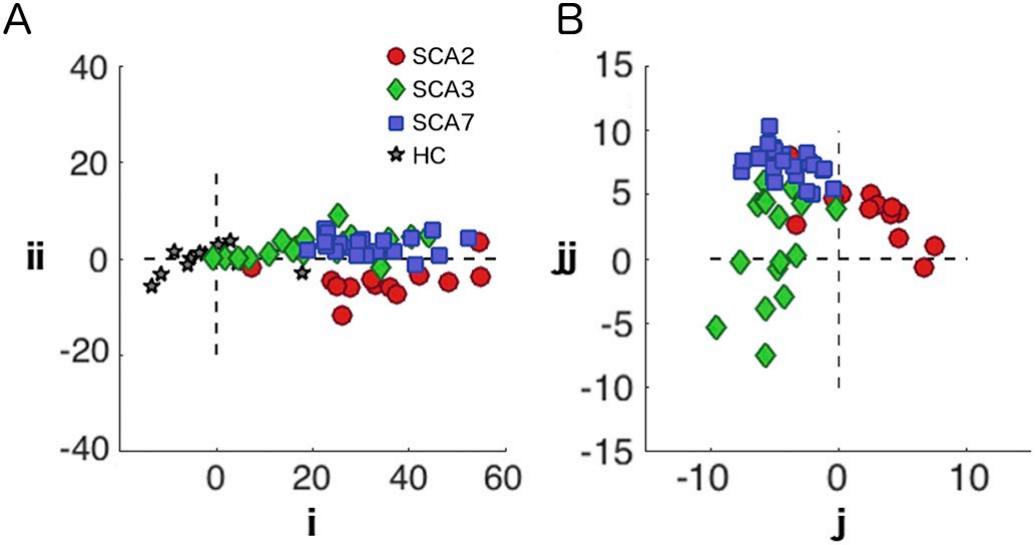
Multidimensional scaling of individual patterns of degeneration. Each marker represents a healthy control (star), a SCA2 patient (red circle), a SCA3 patient (green rhombus) or a SCA7 patient (blue square). The axes of the plot are defined by the first two dimensions of MDS for A) before the severity correction; B) after the severity correction. (For interpretation of the references to color in this figure legend, the reader is referred to the web version of this article.)

To study whether the patterns of degeneration in the cerebellar cortex differed across subtypes, we applied classical multidimensional scaling (MDS) to the scaled gray-matter intensity maps. The axes of the resultant plots (Fig. 3) are defined by the multivariate dimensions (patterns) that explain most of the between-patient differences. Fig. 3a shows the arrangement of the different degeneration patterns before correction for overall severity. The first dimension, relating to the overall severity of degeneration, accounted for 94.5% of the overall variance. Thus, any statistical test or classification approach would most dominantly reflect this factor. In this visualization, the SCA3 patients are positioned most closely to the control group, corroborating the finding that these patients exhibit the smallest amount of degeneration compared to the other two SCAs.

To measure the separability of the groups, we performed a binary classification analysis between each group pair. The crossvalidated classification accuracies between the patient groups and healthy controls were above 93%. This result was expected since the classifier uses the amount of degeneration between patients and controls as the main classification feature. In contrast, the classification between patient groups was poorer, especially between SCA3 and SCA7 (Table 1). Part of the problem is that the classifier attempts to separate these two groups along the severity dimension (i axis in Fig. 3a), which leads to poor cross-validation performance, as there were no clear differences in the degeneration severity between the SCA3 and SCA7 patients.

**Table 1:** Cross-validated classification accuracies for pairwise group comparisons. The left side of the table shows mean classification accuracy for the pairwise comparison between each subtype and healthy controls, the right half between different subtypes. Classification is performed either with or without correction for overall degeneration severity.

Healthy controls vs SCA subtypes		Between SCA subtypes		
Before correction			Before correction	After correction
SCA2	1.000	SCA2-SCA3	0.897	0.899
SCA3	0.932	SCA2-SCA7	0.867	0.905
SCA7	0.954	SCA3-SCA7	0.719	0.931

### 3.5 Separability after severity correction

To ameliorate the problem of severity differences, we normalized each participant's pattern by the overall amount of degeneration (see methods). After severity correction (Fig. 3b), the first two dimensions clearly separated the different subtypes into three distinct clusters.

Correspondingly, the classification accuracies between the patient groups, especially between SCA7 and SCA3, increased substantially (Table 1). Thus, correction for degeneration severity is an important step in testing for differences in degeneration patterns and in ensuring good generalization performance. The high accuracy demonstrates that it is possible to identify a type-specific degeneration pattern signature between different SCAs.

**Fig. 4:**
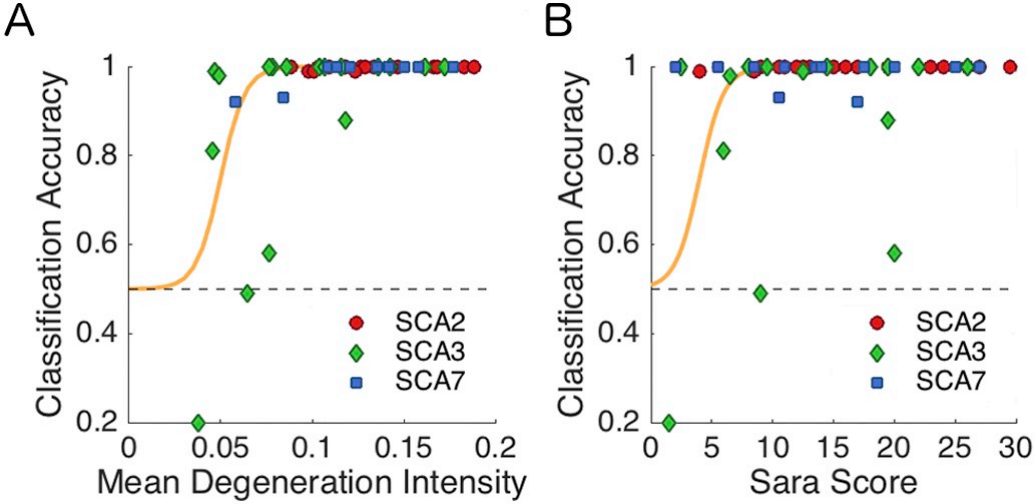
Individual classification accuracy. Cross-validated classification accuracy for each patient, averaged across cross-validation folds and across the three possible pairwise classifications. The accuracy is plotted as a function of A) Mean degeneration severity and B) SARA score (see Methods), with the green line indicating the best logistic fit.

The high classification accuracies suggest that classification based on the pattern of degeneration could serve as an additional diagnostic criterion. One important question for possible clinical applications, however, is whether this classification approach is only successful in differentiating severe patterns of degeneration in later-stage patients, or whether it can also be applied to patients in earlier stages of the disease. To test this, we tracked how well each patient could be classified based on their individual pattern of degeneration. Fig. 4a shows the individual classification accuracy as a function of the mean degeneration severity. For patients with 5% of degeneration, or a SARA score of five and higher, the classifier reached an accuracy of at least 75%. Thus, even for moderate amounts of degeneration, sub-type specific patterns were distinguishable.

### 3.6 Subtype specific degeneration pattern

**Fig. 5:**
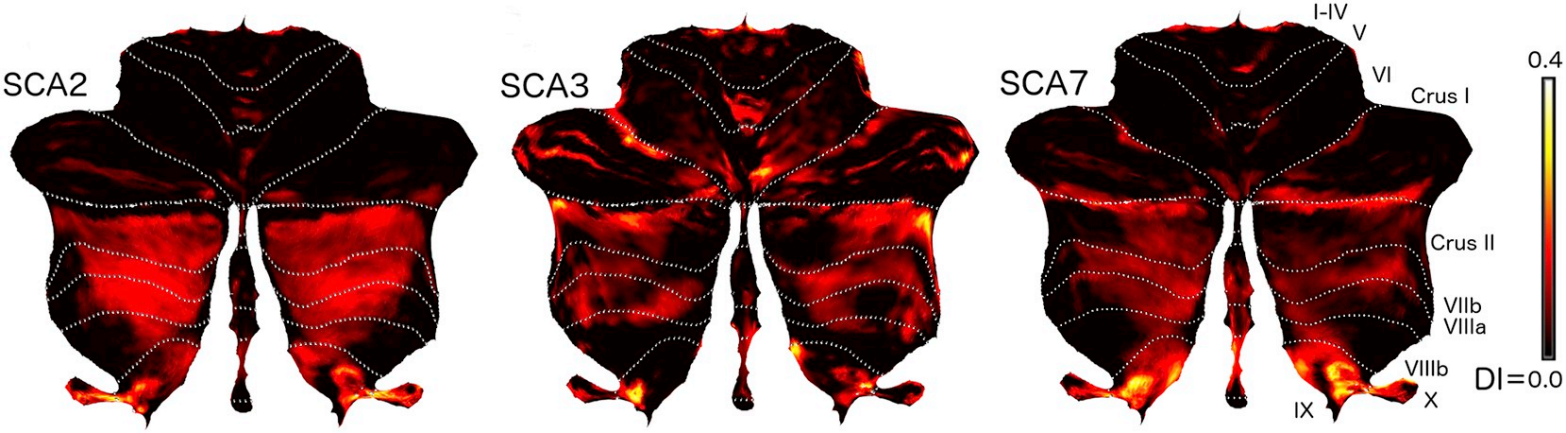
Degeneration signature of each SCA subtype. Cerebellar flat map representation of the degeneration pattern for each SCA. Intensity of color indicates the degeneration of each group (DI = degeneration Intensity).

To understand the differences between the degeneration pattern for the SCA subtypes, we calculated the average degeneration pattern and visualized it on a flat representation of the cerebellum (Fig. 5). Degeneration in SCA2 was restricted mostly to the posterior lobe beginning in crus II and continuing posteriorly to lobule X. In contrast, degeneration in SCA3 also included anterior lobe structures, especially the medial aspects of lobules IV-V. Additionally, lobule VI was more affected as compared to SCA2. The pattern in SCA7 showed less degeneration in the anterior lobe than SCA3, and more degeneration in lobule IX.

**Fig. 6:**
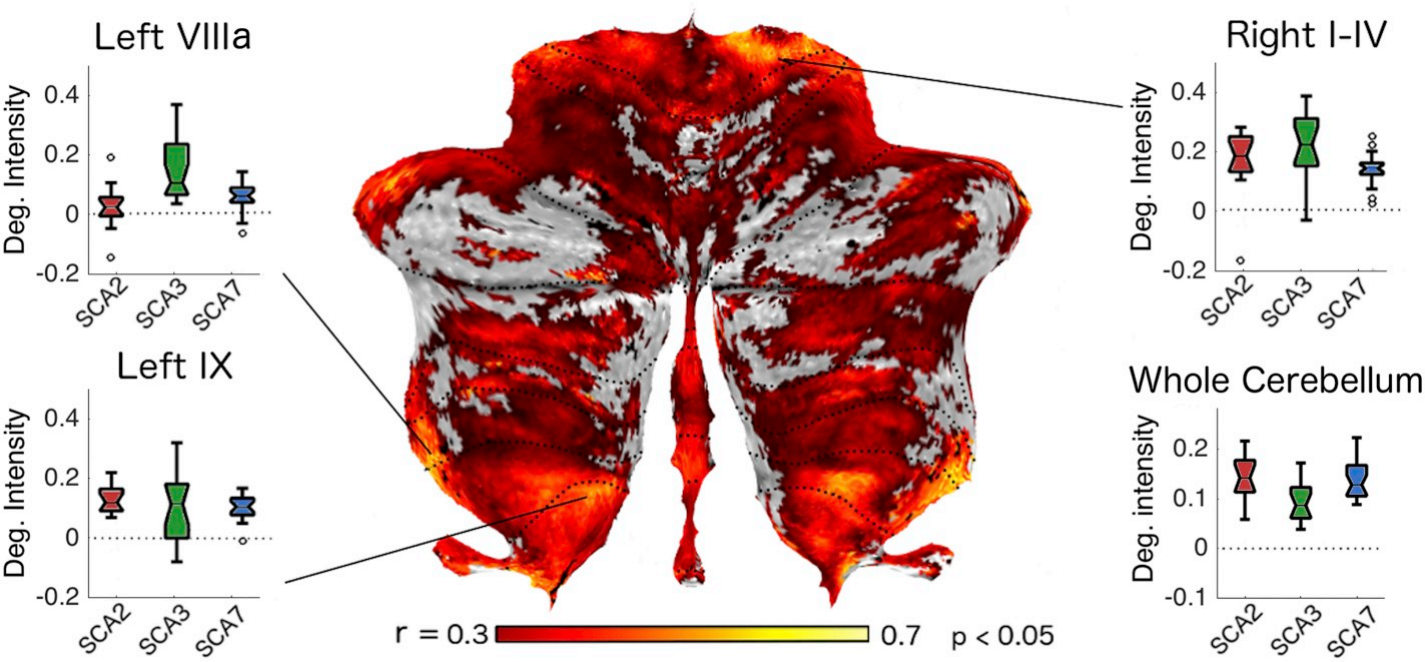
Correlation between SARA score and degeneration intensity. Flat map representation displaying significant correlations between the SARA score and degeneration intensity across all subtypes. Color intensity indicates the Pearson's r value (significance set as *p* < .05). Boxplots indicate the degeneration intensity for each SCA subtype in regions showing higher correlations with SARA score and the mean cerebellar degeneration of each subtype. For a full list of correlation local maxima see Table 2.

We then asked whether these differences in degeneration patterns in the cerebellar cortex could explain the differences in ataxic symptomatology between subtypes. This was of particular interest for the SCA3 subtype, as this group, on average, demonstrated less degeneration for the same impairment score. Specifically, we hypothesized that SCA3 patients would show equal or larger degeneration in areas specifically associated with ataxic symptoms. To test this idea, we first explored the relationship in the patterns and the clinical ataxia score across subtypes. We calculated the Pearson's correlation between the SARA score and the degeneration maps (scale by brain size, but not corrected for overall degeneration severity) across all patients (Fig. 6). This analysis revealed cerebellar regions that were related to the ataxia impairment, e.g. more degeneration in these regions was associated with higher ataxia scores. We found that the SARA score correlated with gray-matter intensity to varying degrees. Clusters with high correlations were found in motor-related regions of the cerebellum, such as lobules I-IV, VIIIa, and VIIIb. Smaller clusters were also visible in areas of the cerebellum that are thought to be involved in non-motor process, such as the lobules IX, Crus I, and the posterior vermis.

To test whether SCA3 patients showed especially high degeneration in areas that are commonly associated with strong motor symptoms, we identified the 10 local maxima on the correlation map. We then compared the amount of degeneration in a 6 mm sphere around these locations across the different subtypes (Table 2). Indeed, in three of the ten regions the amount of degeneration in SCA3 patients significantly exceeded the degeneration in the other two subtypes. This was the case in left lobule VIIIb, right Crus I, and right lobule IV. There were no significant differences in the other six regions and only two regions showed less degeneration. This anatomical differentiation could explain why SCA3 patients showed the same ataxic impairment with significantly less overall degeneration than the other two subtypes. Our results clearly show that the SCA3 mutation affects motor areas more selectively than SCA2 and SCA7.

**Table 2:** Degeneration intensity differences between SCA3 and the other two SCAs. The regions were defined by 10 mm sphere, centered on the local maxima of the map of correlation with SARA score (Fig. 6).

Side	Cerebellar lobule	t(53) of SCA3 vs. (SCA2/SCA7)	*p*-Value
Left	VIIIa/VIIIb/X	2.949	0.004
Right	Crus I	2.534	0.014
Right	I-IV	2.319	0.024
Left	IX	1.741	0.087
Left	VIIIb	−0.436	0.664
Right	VI	−0.649	0.518
Right	IX	−1.263	0.212
Left	Crus II	−1.865	0.067
Right	Crus II	−2.129	0.038
Right	VIIIb	−2.75	0.008

## 4 Discussion

Spinocerebellar ataxias are caused by specific gene mutations that are hypothesized to selectively affect different cerebellar regions. In a careful, quantitative analysis we show for the first time that the degeneration patterns in the cerebellar cortex differ significantly between the SCA2, SCA3, and SCA7 subtypes. Specifically, degeneration was clearly localized to lobules VII-IX in SCA2, but additionally affected lobules VI and medial anterior lobe (IV-V) in SCA3, and lobules IX-X in SCA7. The three SCAs considered here are also known as poly-Q ataxias (Manto, 2005), as they are caused by an abnormal CAG expansion that codes specific proteins called ataxins (Epstein and Martin, 1999). The resulting proteins of each subtype are not homologous. While the normal function of ataxins is currently unknown, our results suggest that specific locations across the cerebellar cortex have different vulnerabilities to different ataxin mutations, resulting in distinct degeneration patterns.

The differences in the degeneration patterns were clear enough that we were able to predict the genetic subtype for individual patients even in earlier stages of the disease. The high cross-validated classification accuracies demonstrate that there is a distinct degeneration signature for each subtype, even when moderately-affected patients are considered. It also suggests that with more advanced analysis tools, non-invasive neuroimaging protocols could be used for differential diagnosis between different types of cerebellar ataxia.

### 4.1 Relationship to ataxia symptoms

Differences in patterns of degeneration across the cerebellar surface were related to differences in the behavioral symptoms of each patient group. It is well known that symptoms can vary in expression and severity across SCAs (Koeppen, 1998) and this can be related to the amount of degeneration, i.e. the cerebellar degeneration in SCA3 appear in the later stages of the disease compared to SCA2 in which atrophy is prominent really early (Koeppen, 2002). The main behavioral measure that was acquired for this patient group was the SARA score, which measures the severity of ataxic motor symptoms. The SARA score was well-matched across the different subtypes in our sample (Fig. 1b), even though the SCA3 patients showed less overall cerebellar degeneration. Indeed, some SCA3 patients had such small amounts of degeneration that a classifier could not accurately distinguish them from healthy controls. One possible scenario that might explain this “early” expression of ataxic symptoms could be that the SCA3 mutation affects the dentate, olivary, and pontine nuclei more so than the other two subtypes. However, in our dataset, the degeneration of brainstem and deep cerebellar nuclei was also less severe in the SCA3 group as compared to the other groups (Fig. 2). Thus, degeneration in cerebellar input and output nuclei does not seem to account for the different symptomology.

Instead, the degeneration in SCA3 could strongly affect motor regions of the cerebellar cortex, which would result in more severe ataxic impairments at earlier stages of the disease. Indeed, we show that motor regions were relatively more impacted in SCA3, likely explaining the earlier expression of ataxic symptoms (Fig. 6). We also demonstrate that it is degeneration in these regions (but not in other parts of the cerebellar cortex) that correlates with the severity of the ataxic symptoms.

In general, differences in the localization of degeneration may be predictive of the different symptom constellations in different cerebellar diseases. For instance, a number of studies have reported cognitive impairments in patients with SCA2, which are not commonly reported in other SCA subtypes (Bürk et al., 2003; Hernandez-Castillo et al., 2015). This could be accounted for by the fact that these patients display extensive degeneration of lobules VII; a region of the cerebellum known to be related to cognitive processing (Schmahmann and Sherman, 1998). Indeed, a comprehensive study correlating cognitive deficits with cerebellar degeneration suggested a close relationship between degeneration in lobules VII and diverse cognitive functions such as semantic and phonetic fluency, working memory, and perceptual organization among others (Kansal et al., 2016; King et al., 2017).

### 4.2 What causes the difference in degeneration patterns?

The physiological underpinnings of the different degeneration patterns are currently unknown. However, a strength of our method is that it allows us to visualize the full degeneration pattern without being constrained by lobular boundaries. Therefore, we can investigate the degree to which the degeneration patterns are determined by the anterior-to-posterior or medial-to-lateral compartmentation of the cerebellum. Animal studies using zebrin II/adolese C (Brochu et al., 1990; Sugihara and Shinoda, 2004) and HSP25 (Armstrong and Hawkes, 2000) staining suggest a medial-to-lateral organization into a series of zebrin positive and zebrin negative zones. It is possible that different degeneration subtypes may differ in their propensity to affect different zones. Following this idea, the more pronounced degeneration in lobules VII-X in SCA2 may be explained by a higher susceptibility of zebrin positive zones to this specific mutation. To test this idea more rigorously, we would require a detailed mapping of the human cerebellum in terms of zones. Even though it has been suggested that non-invasive neuroimaging markers may be able to reveal some aspects of this organization (Boillat et al., 2018), a zebrin map for the human cerebellum is still missing.

Overall, the patterns of degeneration observed in our patients do not follow a clear medial-to-lateral pattern, which would suggest clear zonal differences. Of course, the exact zonal organization in the human cerebellum has yet to be studied in detail. Furthermore, individual and anatomical variability as well as limitations of the VBM methods may obscure the presence of a true relationship between degeneration patterns and zebrin zones.

### 4.3 Clinical application of the degeneration signature

Here, we show that it is possible to classify different SCA subtypes with high accuracy when correcting for the severity of degeneration. This finding suggests that the degeneration signature is a reliable biomarker for the three studied SCA subtypes, even at earlier stages of the disease process. Molecular tests to diagnose SCAs are becoming cheaper and accessible. However, while genetic tests are available for a number of SCAs, some subtypes can only be diagnosed based on their phenotypic expression (Sun et al., 2016). In these cases, the diagnosis can often be challenging, as the symptomatic manifestations of different SCA subtypes show considerable overlap. The methodological advance presented here provides an important first step in the development of an additional tool for the accurate diagnosis of different SCA subtypes. Even if the degeneration is caused by a subtype that can be genetically test, an MRI scan could be conducted prior to molecular testing and would be useful for narrowing the scope of subtype-testing.

### 4.4 Degeneration patterns and trajectories

**Supplementary Fig. 1:**
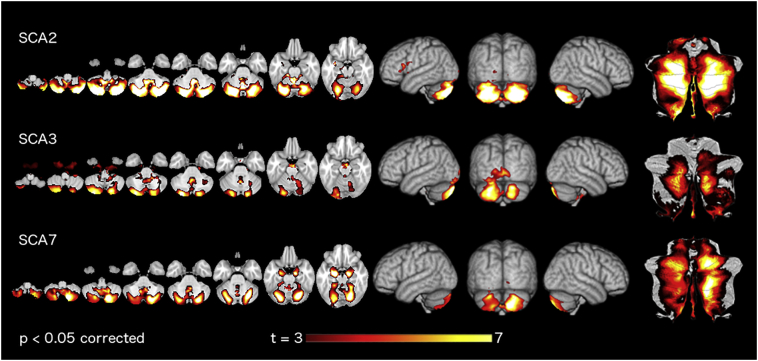
Degeneration pattern of SCAs by regular Voxel-based morphometry. For each SCA group, we applied FSL-VBM. Hot colors indicate the regions where the patients showed a significantly lower gray matter intensity compared to healthy controls. Parametric maps are shown in three different representations (left to right) axial slices, volume rendering, and flat-map.

In more general terms, our work highlights the importance of considering the *pattern* of degeneration independent from the overall *amount* of degeneration. When using regular VBM or volumetric approaches, different groups are usually compared in terms of their absolute gray-matter or volume reduction in specific areas of the brain. While this approach is useful in distinguishing a patient group from a healthy control group, it may not be the optimal for distinguishing groups with different degeneration patterns from each other (for a comparison of regular VBM in this data set see Supplementary Fig. 1). Rather, by correcting the degeneration maps for the overall amount of degeneration, we were able to increase the accuracy of the subtype classification and could thus glean novel insights into the spatial patterns of the degenerative process.

A similar approach may be useful in diagnosing other neurodegenerative diseases, some examples being mild cognitive impairment (MCI), Alzheimer's disease, along with other forms of vascular dementia. Current classification algorithms based on anatomical MRI images alone perform at around 80% when classifying between Alzheimer's and healthy controls (Gerardin et al., 2009; Kloppel et al., 2008; Lao et al., 2004; Westman et al., 2011). However, the classification accuracy is reduced to approximately 20% when trying to predict the onset of Alzheimer's disease from MCI (Zhang et al., 2011). The use of severity-corrected degeneration maps could provide an additional opportunity to increase predictive performance.

Progression of a degenerative disease can be thought of as a trajectory in the space of degeneration patterns as shown in Fig. 3. The direction of the trajectory indicates the specific degeneration pattern associated with the degenerative disease, and the distance along the trajectory would indicate the overall severity. Longitudinal anatomical data now allow for the analysis of individual degeneration trajectories, therefore, a detailed characterization of the speed, direction, and possible curvature of these trajectory may provide novel scientific insights into the disease process, as well as improving clinical prediction. Furthermore, analysis of degeneration trajectories constitutes a potentially important tool for assessing the performance of clinical interventions.

### 4.5 Limitations of the current work

Here, we report that three of the most common SCAs exhibit different degeneration patterns and can be classified with high accuracy based on anatomical data alone. However, an important limitation of this study is that the volumetric analysis of the brainstem was done using traditional methods, using an ROI (combining gray and white matter) from a standardized atlas template. This methodology provided important information regarding the overall amount of degeneration in these regions. However, limitations of current methods prevent us from defining a specific pattern of degeneration within each of the nuclei as we have done in the cerebellar cortex. A challenge for future studies is to improve imaging and analysis techniques that can accurately identify and automatically segment different brainstem nuclei. Such information should then be incorporated into the characterization of the degeneration pattern. Furthermore, we also did not include extracerebellar degeneration such cortical or spinal cord lesions. Different SCAs including the subtypes we analyzed in this work show degeneration in different cortical areas, a more extensive analysis of degeneration in these areas disserves further research. Another limitation is that we were limited to 3 common SCA subtypes. Since SCA is a heterogeneous disease with numerous subtype expressions, the addition of other SCAs (not limited to PolyQ subtypes) would likely reveal other distinctive patterns of degeneration. Furthermore, the extension of our work to patients with idiopathic cerebellar degeneration could yield clusters of consistent degeneration patterns within this heterogeneous group. While our sample size (55 patients) was relatively large, the acquisition of larger cohorts via multicenter collaborations would advance our understanding of spinocerebellar ataxias and other cerebellar diseases.

## 5 Conclusion

Our work provides three novel insights, 1) each of the three studied SCA subtypes presents a unique degeneration pattern in the cerebellar cortex, 2) differences in degeneration patterns can be related to differences in symptomology, and 3) these degeneration signatures can be used to differentiate among subtypes with high accuracy. The methodology employed here allowed for the accurate identification of specific degeneration *patterns*, which could inform us about the possible target location(s) of the degenerative processes underlying clinically heterogeneous neurological diseases.

The following are the supplementary data related to this article.

Supplementary Table 1 Demographic information. Supplementary Table 1

## References

[bb0005] Fisher R.A. (1938). The statistical utilization of multiple measurements. Ann. Eugen.

[bb0010] Goel G., Pal P.K., Ravishankar S., Venkatasubramanian G., Jayakumar P.N., Krishna N. (2011). Gray matter volume deficits in spinocerebellar ataxia: an optimized voxel based morphometric study. Parkinsonism Relat. Disord..

[bb0015] Armstrong C.L., Hawkes R. (2000). Pattern formation in the cerebellar cortex. Biochem. Cell Biol..

[bb0020] Ashburner J. (2007). A fast diffeomorphic image registration algorithm. NeuroImage.

[bb0025] Ashburner J., Friston K.J. (2000). Voxel-based morphometry—the methods. NeuroImage.

[bb0030] Boillat Y., Bazin P.-L., O'Brien K., Fartaria M.J., Bonnier G., Krueger G. (2018). Surface-based characteristics of the cerebellar cortex visualized with ultra-high field MRI. NeuroImage.

[bb0035] Brochu G., Maler L., Hawkes R. (1990). Zebrin II: a polypeptide antigen expressed selectively by purkinje cells reveals compartments in rat and fish cerebellum. J. Comp. Neurol..

[bb0045] Bürk K., Globas C., Bösch S., Klockgether T., Zühlke C., Daum I. (2003). Cognitive deficits in spinocerebellar ataxia type 1, 2, and 3. J. Neurol..

[bb0055] D'Abreu A., França M.C., Yasuda C.L., Campos B.A.G., Lopes-Cendes I., Cendes F. (2012). Neocortical Atrophy in Machado-Joseph Disease: a Longitudinal Neuroimaging Study. J. Neuroimaging.

[bb0060] Della Nave R., Ginestroni A., Tessa C., Cosottini M., Giannelli M., Salvatore E. (2008). Brain structural damage in spinocerebellar ataxia type 2. A voxel-based morphometry study. Mov. Disord..

[bb0065] Diedrichsen J. (2006). A spatially unbiased atlas template of the human cerebellum. NeuroImage.

[bb0070] Diedrichsen J., Zotow E., Pakkenberg B., Donchin O., Hermsdorfer J., Gizewski E. (2015). Surface-based display of volume-averaged cerebellar imaging data. PLoS One.

[bb0075] Dueñas A.M., Goold R., Giunti P. (2006). Molecular pathogenesis of spinocerebellar ataxias. Brain.

[bb0080] Epstein F.H., Martin J.B. (1999). Molecular basis of the neurodegenerative disorders. N. Engl. J. Med..

[bb0085] Everett C.M., Wood N.W. (2004). Trinucleotide repeats and neurodegenerative disease. Brain.

[bb0090] Fahl C.N., Branco L.M.T., Bergo F.P.G., D'Abreu A., Lopes-Cendes I., França M.C. (2015). Spinal cord damage in Machado-Joseph disease. Cerebellum.

[bb0095] Gerardin E., Chételat G., Chupin M., Cuingnet R., Desgranges B., Kim H.-S. (2009). Multidimensional classification of hippocampal shape features discriminates Alzheimer's disease and mild cognitive impairment from normal aging. NeuroImage.

[bb0100] Good C.D., Johnsrude I.S., Ashburner J., Henson R.N., Friston K.J., Frackowiak R.S. (2001). A voxel-based morphometric study of ageing in 465 normal adult human brains. NeuroImage.

[bb0105] Hernandez-Castillo C.R., Alcauter S., Galvez V., Barrios F.A., Yescas P., Ochoa A. (2013). Disruption of visual and motor connectivity in spinocerebellar ataxia type 7. Mov. Disord..

[bb0110] Hernandez-Castillo C.R., Galvez V., Mercadillo R.E., Díaz R., Yescas P., Martinez L. (2015). Functional connectivity changes related to cognitive and motor performance in spinocerebellar ataxia type 2. Mov. Disord..

[bb0115] Hernandez-Castillo C.R., Galvez V., Diaz R., Fernandez-Ruiz J. (2016). Specific cerebellar and cortical degeneration correlates with ataxia severity in spinocerebellar ataxia type 7. Brain Imaging Behav..

[bb0120] Hernandez-Castillo C.R., Diaz R., Campos-Romo A., Fernandez-Ruiz J. (2017). Neural correlates of ataxia severity in spinocerebellar ataxia type 3/Machado-Joseph disease. Cerebellum & Ataxias.

[bb0125] Jacobi H., Reetz K., du Montcel S.T., Bauer P., Mariotti C., Nanetti L. (2013). Biological and clinical characteristics of individuals at risk for spinocerebellar ataxia types 1, 2, 3, and 6 in the longitudinal RISCA study: analysis of baseline data. Lancet Neurol..

[bb0130] Kansal K., Yang Z., Fishman A.M., Sair H.I., Ying S.H., Jedynak B.M. (2016). Structural cerebellar correlates of cognitive and motor dysfunctions in cerebellar degeneration. Brain.

[bb0135] King M., Hernandez-Castillo C., Diedrichsen J. (2017). Towards a multi-function mapping of the cerebellar cortex. Brain.

[bb0140] Klaes A., Reckziegel E., Franca M.C., Rezende T.J.R., Vedolin L.M., Jardim L.B. (2016). MR Imaging in Spinocerebellar Ataxias: a Systematic Review. Am. J. Neuroradiol..

[bb0145] Kloppel S., Stonnington C.M., Chu C., Draganski B., Scahill R.I., Rohrer J.D. (2008). Automatic classification of MR scans in Alzheimer's disease. Brain.

[bb0150] Koeppen A.H. (1998 Jun 1). The hereditary ataxias. J. Neuropathol. Exp. Neurol..

[bb0155] Koeppen A.H. (2002). Neuropathology of the Inherited Ataxias. The Cerebellum and its Disorders.

[bb0160] Lao Z., Shen D., Xue Z., Karacali B., Resnick S.M., Davatzikos C. (2004). Morphological classification of brains via high-dimensional shape transformations and machine learning methods. NeuroImage.

[bb0165] Manto M.-U. (2005). The wide spectrum of spinocerebellar ataxias (SCAs). Cerebellum.

[bb0175] Orr H.T., Zoghbi H.Y. (2007). Trinucleotide repeat Disorders. Annu. Rev. Neurosci..

[bb0180] Orr H.T., Chung M., Banfi S., Kwiatkowski T.J., Servadio A., Beaudet A.L. (1993). Expansion of an unstable trinucleotide CAG repeat in spinocerebellar ataxia type 1. Nat. Genet..

[bb0185] Pulst S.-M., Nechiporuk A., Nechiporuk T., Gispert S., Chen X.-N., Lopes-Cendes I. (1996). Moderate expansion of a normally biallelic trinucleotide repeat in spinocerebellar ataxia type 2. Nat. Genet..

[bb0190] Reetz K., Costa A.S., Mirzazade S., Lehmann A., Juzek A., Rakowicz M. (2013). Genotype-specific patterns of atrophy progression are more sensitive than clinical decline in SCA1, SCA3 and SCA6. Brain.

[bb0195] de Rezende T.J.R., D'Abreu A., Guimarães R.P., Lopes T.M., Lopes-Cendes I., Cendes F. (2015). Cerebral cortex involvement in Machado−Joseph disease. Eur. J. Neurol..

[bb0200] Schmahmann J.D., Sherman J.C. (1998). The cerebellar cognitive affective syndrome. Brain.

[bb0205] Schmitz-Hübsch T., du Montcel S.T., Baliko L., Berciano J., Boesch S., Depondt C. (2006). Scale for the assessment and rating of ataxia: development of a new clinical scale. Neurology.

[bb0210] Schulz J.B., Borkert J., Wolf S., Schmitz-Hübsch T., Rakowicz M., Mariotti C. (2010). Visualization, quantification and correlation of brain atrophy with clinical symptoms in spinocerebellar ataxia types 1, 3 and 6. Neuroimage.

[bb0215] Soong B., Paulson H.L. (2007). Spinocerebellar ataxias: an update. Curr. Opin. Neurol..

[bb0220] Sugihara I., Shinoda Y. (2004). Molecular, topographic, and functional organization of the cerebellar cortex: a study with combined aldolase C and olivocerebellar labeling. J. Neurosci..

[bb0225] Sun Y.-M., Lu C., Wu Z.-Y. (2016). Spinocerebellar ataxia: relationship between phenotype and genotype - a review. Clin. Genet..

[bb0230] Torgerson W.S. (1952). Multidimensional scaling: I. Theory and method. Psychometrika.

[bb0235] Westman E., Simmons A., Zhang Y., Muehlboeck J.-S., Tunnard C., Liu Y. (2011). Multivariate analysis of MRI data for Alzheimer's disease, mild cognitive impairment and healthy controls. NeuroImage.

[bb0240] Yamada M. (2013). Neuropathology of Ataxias. Handbook of the Cerebellum and Cerebellar Disorders.

[bb0245] Zhang D., Wang Y., Zhou L., Yuan H., Shen D. (2011). Multimodal classification of Alzheimer's disease and mild cognitive impairment. NeuroImage.

