# Supplementary material for: Unique degeneration signatures in the cerebellar cortex for spinocerebellar ataxias 2, 3, and 7
Source: Neuroimage Clin. 2018 Sep 27;20:931–8. doi: 10.1016/j.nicl.2018.09.026 (PMC6178193; doi:10.1016/j.nicl.2018.09.026)
Supplement: Supplementary Table 1 — Demographic information. [file mmc1.docx]

**Supplementary table 1**. Demographic information.

| **ID** | **Age** | **SARA** | **Gender** | **Subtype** |
| --- | --- | --- | --- | --- |
| S01 | 29 | 27 | F | SCA2 |
| S02 | 35 | 8.5 | M | SCA2 |
| S03 | 60 | 13.5 | F | SCA2 |
| S04 | 19 | 5.5 | M | SCA2 |
| S05 | 29 | 20 | M | SCA2 |
| S06 | 31 | 17 | F | SCA2 |
| S07 | 58 | 17.5 | M | SCA2 |
| S08 | 20 | 10.5 | M | SCA2 |
| S09 | 48 | 11 | F | SCA2 |
| S10 | 43 | 25 | F | SCA2 |
| S11 | 46 | 14 | F | SCA2 |
| S12 | 20 | 17.5 | M | SCA2 |
| S13 | 41 | 33.5 | F | SCA2 |
| S14 | 65 | 25 | F | SCA2 |
| S15 | 18 | 2 | F | SCA2 |
| S16 | 49 | 26 | F | SCA3 |
| S17 | 58 | 19.5 | M | SCA3 |
| S18 | 45 | 26 | F | SCA3 |
| S19 | 37 | 12.5 | M | SCA3 |
| S20 | 35 | 6.5 | F | SCA3 |
| S21 | 23 | 6 | F | SCA3 |
| S22 | 42 | 8 | F | SCA3 |
| S23 | 24 | 9 | F | SCA3 |
| S24 | 18 | 2.5 | F | SCA3 |
| S25 | 45 | 8 | F | SCA3 |
| S26 | 43 | 14.5 | M | SCA3 |
| S27 | 34 | 18 | M | SCA3 |
| S28 | 59 | 19.5 | M | SCA3 |
| S29 | 33 | 9.5 | M | SCA3 |
| S30 | 40 | 20 | F | SCA3 |
| S31 | 56 | 14.5 | M | SCA3 |
| S32 | 46 | 1.5 | F | SCA3 |
| S33 | 40 | 27 | F | SCA7 |
| S34 | 44 | 9 | F | SCA7 |
| S35 | 68 | 6 | F | SCA7 |
| S36 | 43 | 15 | F | SCA7 |
| S37 | 42 | 29.5 | F | SCA7 |
| S38 | 18 | 19.5 | M | SCA7 |
| S39 | 39 | 27 | F | SCA7 |
| S40 | 18 | 7 | F | SCA7 |
| S41 | 34 | 17 | M | SCA7 |
| S42 | 35 | 16 | M | SCA7 |
| S43 | 64 | 14.5 | M | SCA7 |
| S44 | 47 | 13 | M | SCA7 |
| S45 | 23 | 12.5 | M | SCA7 |
| S46 | 44 | 11 | M | SCA7 |
| S47 | 40 | 23 | F | SCA7 |
| S48 | 60 | 16 | M | SCA7 |
| S49 | 54 | 24 | M | SCA7 |
| S50 | 45 | 12 | F | SCA7 |
| S51 | 35 | 8.5 | F | SCA7 |
| S52 | 21 | 4 | F | SCA7 |
| S53 | 20 | 4 | M | SCA7 |
| S54 | 30 | 12 | M | SCA7 |
| S55 | 61 | 10.5 | M | SCA7 |
| S56 | 57 | - | M | CONT |
| S57 | 27 | - | M | CONT |
| S58 | 24 | - | M | CONT |
| S59 | 60 | - | M | CONT |
| S60 | 35 | - | F | CONT |
| S61 | 44 | - | F | CONT |
| S62 | 58 | - | F | CONT |
| S63 | 42 | - | F | CONT |
| S64 | 41 | - | F | CONT |
| S65 | 54 | - | M | CONT |
| S66 | 55 | - | F | CONT |
| S67 | 35 | - | M | CONT |
| S68 | 24 | - | M | CONT |
| S69 | 47 | - | F | CONT |
| S70 | 23 | - | M | CONT |
